# Aerobic Capacity, Lactate Concentration, and Work Assessment During Maximum Exercise at Sea Level and High Altitude in Miners Exposed to Chronic Intermittent Hypobaric Hypoxia (3,800 m)

**DOI:** 10.3389/fphys.2019.01149

**Published:** 2019-09-06

**Authors:** Fernando A. Moraga, Jorge Osorio, Daniel Jiménez, Rodrigo Calderón-Jofré, Daniel Moraga

**Affiliations:** ^1^Laboratorio de Fisiología, Hipoxia y Función Vascular, Departamento de Ciencias Biomédicas, Facultad de Medicina, Universidad Católica del Norte, Coquimbo, Chile; ^2^Instituto de Estudios de la Salud, Universidad Arturo Prat, Iquique, Chile; ^3^Escuela de Salud Pública, Facultad de Medicina, Universidad de Chile, Santiago, Chile; ^4^Departamento de Ciencias Básicas, Universidad Santo Tomás, La Serena, Chile; ^5^Carrera de Medicina, Facultad de Ciencias de la Salud, Universidad de Tarapacá, Arica, Chile

**Keywords:** chronic hypobaric intermittent hypoxia, aerobic capacity, lactate concentration, work assessment, miners workers

## Abstract

We previously showed that arterial oxygen content during maximum exercise remains constant at high altitude (HA) in miners exposed to chronic intermittent hypobaric hypoxia (CIHH). Nevertheless, information about VO_2_, lactate concentration [Lac], and work efficiency are absent in this CIHH miner population. Our aim was to determine aerobic capacity, [Lac], and work efficiency at sea level (SL) and HA during maximum exercise in miners acclimatized to CIHH at 3,800 m. Eight volunteer miners acclimatized to CIHH at HA (> 4 years) performed an exercise test at SL and HA. The test was performed on the 4th day at HA or SL and consisted of three phases: Rest (5 min); Exercise test, where the load was increased by 50 W every 3 min until exhaustion; and a Recovery period of 30 min. During the procedure VO_2_, transcutaneous arterial saturation (SpO_2_, %), and HR (bpm) were assessed at each step by a pulse oximeter and venous blood samples were taken to measure [Lac] and hemoglobin concentration. No differences in VO_2_ and [Lac] in SL vs. HA were observed in this CIHH miner population. By contrast, a higher HR and lower SpO_2_ were observed at SL compared with HA. During maximum exercise, a reduction in VO_2_ and [Lac] was observed without differences in intensity (W) and HR. A decrease in [Lac] was observed at maximum effort (250 W) and recovery at HA vs. SL. These findings are related to an increased work efficiency assessment such as gross and net efficiency. This study is the first to show that miners exposed to CIHH maintain their work capacity (intensity) with a fall in oxygen consumption and a decrease in plasmatic lactate concentration at maximal effort at HA. These findings indicate that work efficiency at HA is enhanced.

## Introduction

Exposure to hypoxia conditions, such as high altitude (HA), limits oxygen transport from the air into the muscle, affecting work capacity (Schoene, 2001; Calbet and Lundby, 2009). The same authors propose that oxygen limitation in lung diffusion is responsible for the reduced oxygen consumption at HA. In this sense, HA hypoxia limits many physical tasks that are commonly performed at sea level (SL).

Early studies showed that during acclimatization at HA (2,800–6,140 m), in subjects exposed to progressive exercise, lactate production was reduced at maximum effort in correlation with the altitude reached (Edwards, 1936). Other studies have shown that lactate production does not vary during exercise in acute hypoxia (Hughes et al., 1968). Studies performed in climbers showed that maximum oxygen consumption decreases proportionally with altitude over 5,000 m (West, 1986). Early studies have shown that there is a lower lactate response during maximal exercise and recovery (Sutton et al., 1988; Green et al., 1989), and this response is lower in Andeans and Sherpas than in the lowlands. The term “lactate paradox” was used by West in 1986, it and defines the phenomenon of a progressive decrease in plasma lactate when increasing altitude during maximum effort. In addition, evidence shows that Quechuas (inhabitants of 4,200 m) have a lower lactate response to maximal exercise, which supports the decoupling of lactate production that remains when Quechuas descend to low altitude for 6 weeks (Hochachka et al., 1991, 1992). By contrast, some authors support the evidence that no “lactate paradox” was observed in chronically exposed climbers for 9 weeks at an altitude of 5,260 m (van Hall et al., 2001) nor for 8 weeks at 4,100 m (van Hall et al., 2009).

Over the last 30 years, mining activity in Chile has relocated miners that normally live at SL lowlands to work at HA (>3,000 m). This altitude shift is called the “Chilean model of Chronic Intermittent Hypobaric Hypoxia (CIHH) exposure,” which is characterized by alternating periods of work at HA and rest periods at low altitude <1,000 m (Jiménez, 1995; Richalet et al., 2002; Moraga et al., 2018).

Nowadays, exposure studies on maximal aerobic capacity are scarce in this model. Initial studies with CIHH miners began with a prospective study with CIHH exposure of 36 months at HA (4,500 m), where an inverse relation between the miner’s exposure time with a decrease in physical performance was observed (Richalet et al., 2002). A second study performed in soldiers with CIHH exposure during 6 months at HA (3,550 m), showed a tendency for maximal aerobic capacity (VO_2_ max) to decrease at HA with a maintained oxygen transport capacity (Prommer et al., 2007). A third study performed in miners acclimatized to CIHH for a long time period (7–36 months) showed that the maintained oxygen transport is explained by an increased hemoglobin concentration *pari-passu* with increased intensity (Moraga et al., 2018). Additionally, the same study reported that maximum intensity observed during exercise at SL or HA is not modified. This evidence strongly suggests that when miners are exposed to CIHH, oxygen supply is maintained. However, the literature lacks evidence regarding oxygen capacity, work efficiency, and lactate metabolism in CIHH miners. In this sense, evaluation of oxygen consumption, lactate concentration, and estimation of work efficiency in this population exposed to CIHH are necessary. Our aim was to determine aerobic capacity, work assessment, and lactate concentration at SL and HA (3,800 m) during maximum exercise in miners acclimatized to CIHH.

## Materials and Methods

### Subjects

**Figure 1:**
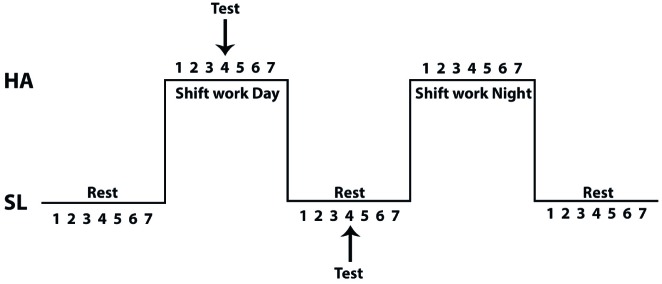
A model of chronic intermittent hypobaric hypoxia exposure. Arrows show day when the evaluations were performed during the shiftwork day at high altitude or sea level.

Twelve voluntary subjects were enrolled in the study; all miners live at low altitude (<1,000 m) and work at HA (3,800 m). All volunteers are heavy truck operators and have experience with CIHH for more than 4 years. Their shift pattern was 7 days of work at HA followed by 7 days resting at SL (Figure 1). All subjects were healthy, non-smokers, and performed moderate physical activity. Protocols used in this study were in accordance with International ethical guidelines (according to the Helsinki declaration) and approved by the Ethics Committee of the Facultad de Medicina of the Universidad Católica del Norte, Chile and Medical Direction of miner company. All volunteers read about the possible risks and discomfort involved before giving and their signed consent to participate.

### Metabolic and Work Assessment

All tests were performed at day 4 of the shift work at HA and at day 4 of the shift work at SL (Figure 1). The study considered two maximum exercise tests, one at SL (in our laboratory) and the other at HA (in a clinic located in the mining complex at 3,800 m). Exercise was performed on a cycle ergometer (Model Corival, Lode) where oxygen consumption and ventilator variables were determined using a metabolic cart (Ultima CPX, Medgraphics, St. Paul, Minnesota, USA) calibrated prior to each test according to the manufacturer’s instructions, with high grade calibration gases (purchased to INDURA, Chile). Respiratory variables were analyzed breath-by-breath in real time and averaged each 5 s during all test. The protocol was similar to that described previously (Moraga et al., 2018) and consisted of three phases: Rest (5 min), Exercise test (increasing the load by 50 W every 3 min until exhaustion, maintaining a cadence of 70 rpm), and Recovery (30 min after exercise). During the protocol, transcutaneous arterial saturation (SpO_2_, %) and heart rate (HR, bpm) were assessed by a pulse oximeter (8500M Nonin Medical, Inc., USA), with the sensor placed on an ear lobe (8000Q2 Nonin Medical, Inc., USA). For lactate and hemoglobin concentration (Hb) and hematocrit measurements, blood samples were taken by intravenous catheter inserted from the brachial vein allowing for blood sample collection during the rest period, at the end of each load (50, 100, 150, 200, and 250 W) during the exercise test and during recovery 30 min after the end of the test. For each sample, 3 ml of venous blood was collected in a tube with EDTA. Blood samples were centrifuged immediately after collection (3,000 rpm for 20 min), plasma fraction was frozen in liquid nitrogen. Afterward, plasma samples were stored at −80°C until perform assay.

### Blood Analysis

Lactate concentration ([Lac]) was determined by spectrophotometry using a commercial kit (Lactate Assay Kit II, Sigma Aldrich), according to the manufacturer’s specifications, and values were expressed as mmol/L. Hematological determinations were made using a hematological counter (Cell-Dyn 1400, Abbott) and hemoglobin concentration (g/dl) was measured. Oxygen content (CaO_2_) was calculated using the following equation: CaO_2_ (ml O_2_/dl) = 1.34 (ml O_2_/gHb) × Hb (g/dl) × SpO_2_ (%) (Calbet et al., 2015; Moraga et al., 2018), where the maximum volume of oxygen that combines with 1 g of hemoglobin is 1.34.

### Work Assessment

*Gross efficiency* (%) was defined as the ratio of work accomplished divided by the energy expended multiplied by 100. *Net efficiency* (%) was defined as the ratio between work accomplished divided by energy expended at rest multiplied by 100. To calculate values of *gross efficiency* or *net efficiency*, values of oxygen consumption were converted to W/kg and intensity was expressed in W/kg (Gaesser and Brooks, 1975; Hochachka et al., 1991).

### Statistical Analysis

Data analysis were performed using only 8 of the initially enrolled 12 subjects, because only eight of these volunteers finalized all tests at SL and HA. Values presented in tables and figures represent paired values of each volunteer at SL or HA, and these values were expressed as the mean ± SD. Statistical analysis was performed with GraphPad Prism Software (version 5.03, GraphPad Software, Inc.). Significant differences were analyzed using ANOVA followed by a Newman-Keuls test. To evaluate the significant differences between HA and SL, paired *t*-test and Pearson correlation were performed. Values of *p* < 0.05 were considered statistically significant.

## Results

### Resting Values at Sea Level and High Altitude Conditions

Characteristics of the voluntaries in rest condition are shown in Table 1. Volunteers are slightly overweight. Hemoglobin concentration, hematocrit, and HR was significantly higher in the HA condition than the SL condition (*p* < 0.05). By contrast, oxygen saturation was significantly lower in the HA condition than the SL condition (*p* < 0.05). No significant difference was observed between the SL and HA conditions in plasma lactate concentration at rest (Table 1).

**Table 1:** Characteristics of the study group.

	Sea level (0 m)	High altitude (3,800 m)
Age (years)	32.3 ± 1.0	
Weight (kg)	75.5 ± 8.3	
Height (m)	1.72 ± 0.1	
Body mass index (kg/m^2^)	25.5 ± 2.2	
Hb (g/dl)	14.2 ± 0.4	15.9 ± 0.4^*^
Hematocrit (%)	44.3 ± 1.2	46.1 ± 1.5^*^
Heart rate (bpm)	71.3 ± 8.3	84.2 ± 9.1^*^
Oxygen saturation (%)	97.5 ± 0.8	92.3 ± 1.3^*^
[Lac] (mmol/L)	1.1 ± 0.2	1.2 ± 0.3

*Values are expressed as mean ± SD*.

* *A significant difference between sea level and high altitude (*p* < 0.05)*.

### Cardiorespiratory and Metabolic Test Performed During Maximum Exercise at Sea Level and High Altitude Conditions

Resting and maximal values of cardiorespiratory and metabolic variables after exercise in SL and HA conditions are shown in Table 2. Significant differences between resting and maximum exercise were found between rest and maximal exercise, except for P_ET_CO_2_, CaO_2_ (in SL and HA), and P_ET_O_2_ and hemoglobin concentration (only HA).

**Table 2:** Summary of cardiorespiratory and metabolic values at rest and maximum exercise.

	Sea level (0 m)		High altitude (3,800 m)	
	Rest	Maximum	Rest	Maximum
Intensity (W)	0	250	0	250
VO_2_ (L/min)	0.37 ± 0.08	2.82 ± 0.46^*^	0.29 ± 0.09	1.99 ± 0.47^*^^,^^†^
VCO_2_ (L/min)	0.35 ± 0.04	3.37 ± 0.39^*^	0.21 ± 0.08^†^	2.13 ± 0.3^*^^,^^†^
RER	0.93 ± 0.2	1.2 ± 0.07^*^	0.78 ± 0.15	1.09 ± 0.21^*^
P_ET_O_2_ mmHg	111 ± 1.1	116.3 ± 2.1	67.2 ± 4.7^†^	72.6 ± 4.9^†^
P_ET_CO_2_ (mmHg)	34.3 ± 2.9	32.8 ± 2.4	18.9 ± 0.9^†^	19.2 ± 4.7^†^
RR (bpm)	14.0 ± 4.7	39.3 ± 9^*^	14.7 ± 5.3	47.4 ± 13.8^*^
*V*_E_ (L/min)	14.0 ± 3.1	122.8 ± 18.5^*^	18.8 ± 5.4	159.9 ± 23^*^^,^^†^
SpO_2_ (%)	97.5 ± 1	93.2 ± 2.1^*^	93.0 ± 2.7^†^	85.6 ± 3.3^*^^,^^†^
Hb (g/dl)	15.3 ± 0.3	16.0 ± 0.5	16.1 ± 0.3^†^	17.2 ± 0.7^*^
CaO_2_ (ml O_2_/dl)	14.9 ± 0.8	15.9 ± 0.8	14.9 ± 0.6	14.7 ± 1.2
HR (bpm)	71.3 ± 13	172.8 ± 10^*^	84.2 ± 13^†^	178.6 ± 17^*^
[Lac] (mmol/L)	1.6 ± 0.4	13.2 ± 1.3^*^	1.5 ± 0.4	11.1 ± 0.8^*^^,^^†^

*Values expressed as mean ± SD*.

* *A significant difference between rest and maximum*.

† S*ignificant difference between sea level and high altitude (*p* < 0.05)*.

In SL and HA, *V_E_* increased by 8.8 and 8.5 times, respectively (*p* < 0.05). This increase can be partially explained by an increase in RR (2.4 times at SL and 3.2 times at HA) and an increase in the tidal volume (3.1 times at SL and 3.4 times at HA). When we express values such as ventilation divided by VO_2_ (V_E_/VO_2_), a significant increase in the respiratory stimulus could be observed at HA in comparison with SL conditions (84.8 ± 4.0 and 43.8 ± 5.1, *p* < 0.05, respectively) and ventilation divided by VCO_2_ (V_E_/VCO_2_) obtained values of 76.7 ± 0.9 and 36.5 ± 3.6, *p* < 0.05, respectively. The heart rate response during maximal exercise showed no differences between SL and HA. The oxygen saturation response at maximal exercise performed at SL showed a significant decrease in oxygen saturation (*p* < 0.05) with a maintained hemoglobin concentration and oxygen content. By contrast, in the HA condition, the decrease in the oxygen saturation is higher than SL, and an increase in the hemoglobin concentration (*p* < 0.05) and a maintained oxygen content was observed (Table 2).

**Figure 2:**
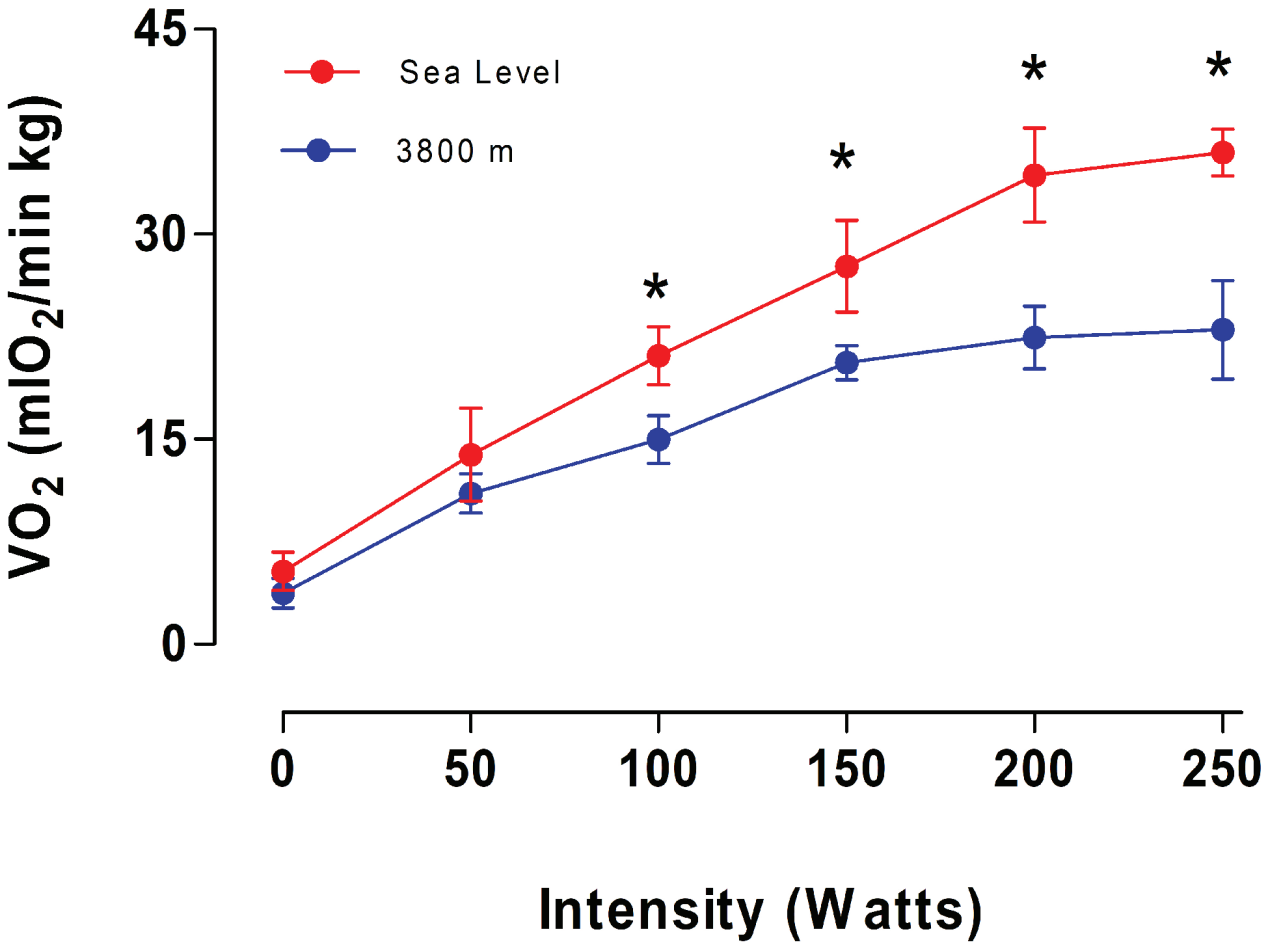
Average values of oxygen consumption (VO_2_) as a function of intensity (in W) obtained on the fourth day at sea level and 3,800 m. * represents a significant difference between Sea level vs. 3,800 m (*p* < 0.05).

Figure 2 shows oxygen consumption during maximum exercise at SL and HA conditions, with no significant difference before an intensity of 50 W. However, at higher intensities (100–250 W), a significant decrease in the oxygen consumption was observed in subjects at HA compared with SL. An average reduction of 29.4% in the maximal oxygen consumption was observed in volunteers at HA in comparison with volunteers at SL at 250 W (*p* < 0.05). These results suggest an increment in work efficiency evaluated at HA compared to the same volunteer at SL.

### Work Efficiency During Maximal Exercise Test Performed at Sea Level and High Altitude Conditions

**Figure 3:**
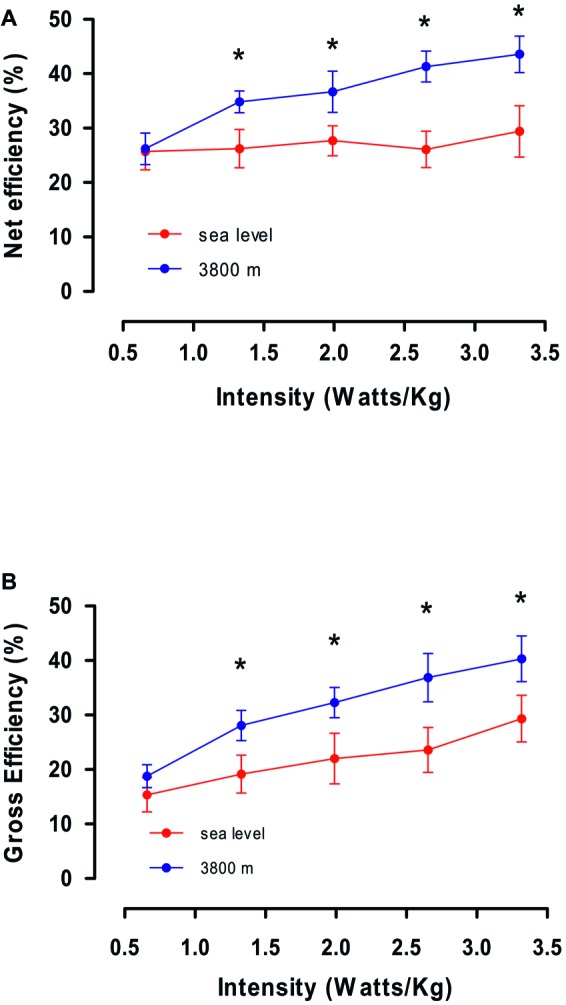
**(A,B)** show average values of calculated Net efficiency and Gross efficiency as a function of intensity (in W/kg) obtained on the fourth day at Sea level and 3,800 m. * represents a significant difference between Sea level vs. High altitude (*p* < 0.05).

In order to explain the minor VO_2_ max obtained at HA compared with SL at the same intensity, we calculated values of *gross efficiency* and *net efficiency* obtained during steady state for exercise performed at SL and HA. In Figure 3A, we observed that calculated *gross efficiency* at HA is higher than *gross efficiency* at SL (37.4 ± 5.2 and 27.3 ± 4.5, *p* < 0.05, Table 3), and this increase is related to the increase in intensity of effort for both conditions. Additionally, in Figure 3B, we observed a higher *net efficiency* at HA compared to SL (44.1 ± 6.4 and 31.1 ± 4.6, *p* < 0.05, Table 3), and *net efficiency* increase with higher intensity of effort (Figure 3B). By contrast, *net efficiency* is not modified by an increase in the effort intensity at SL. The higher *gross* and *net efficiency* observed in volunteers at HA suggests a better mechanism for promoting work maintenance with a lower VO_2_ at intensities over 100 W.

**Table 3:** Work assessment at sea level and high altitude.

	Sea level (0 m)		High altitude (3,800 m)	
	Rest	Maximum	Rest	Maximum
Intensity (W/kg)	0	3.32 ± 0.56	0	3.32 ± 0.56
VO_2_ (W/kg)	1.85 ± 0.4	12.0 ± 1.3^*^	1.7 ± 0.2	9.4 ± 3.7^*^^,^^†^
Gross efficiency (%)	–	27.3 ± 4.5	–	37.4 ± 5.2^†^
Net efficiency (%)	–	31.1 ± 4.6	–	44.1 ± 6.4^†^

*Values expressed as mean ± SD*.

* *A significant difference between rest and maximum*.

† *Significant difference between sea level and high altitude (*p* < 0.05)*.

### Plasma Lactate Concentration During a Maximal Exercise Test Performed at Sea Level and High Altitude

**Figure 4:**
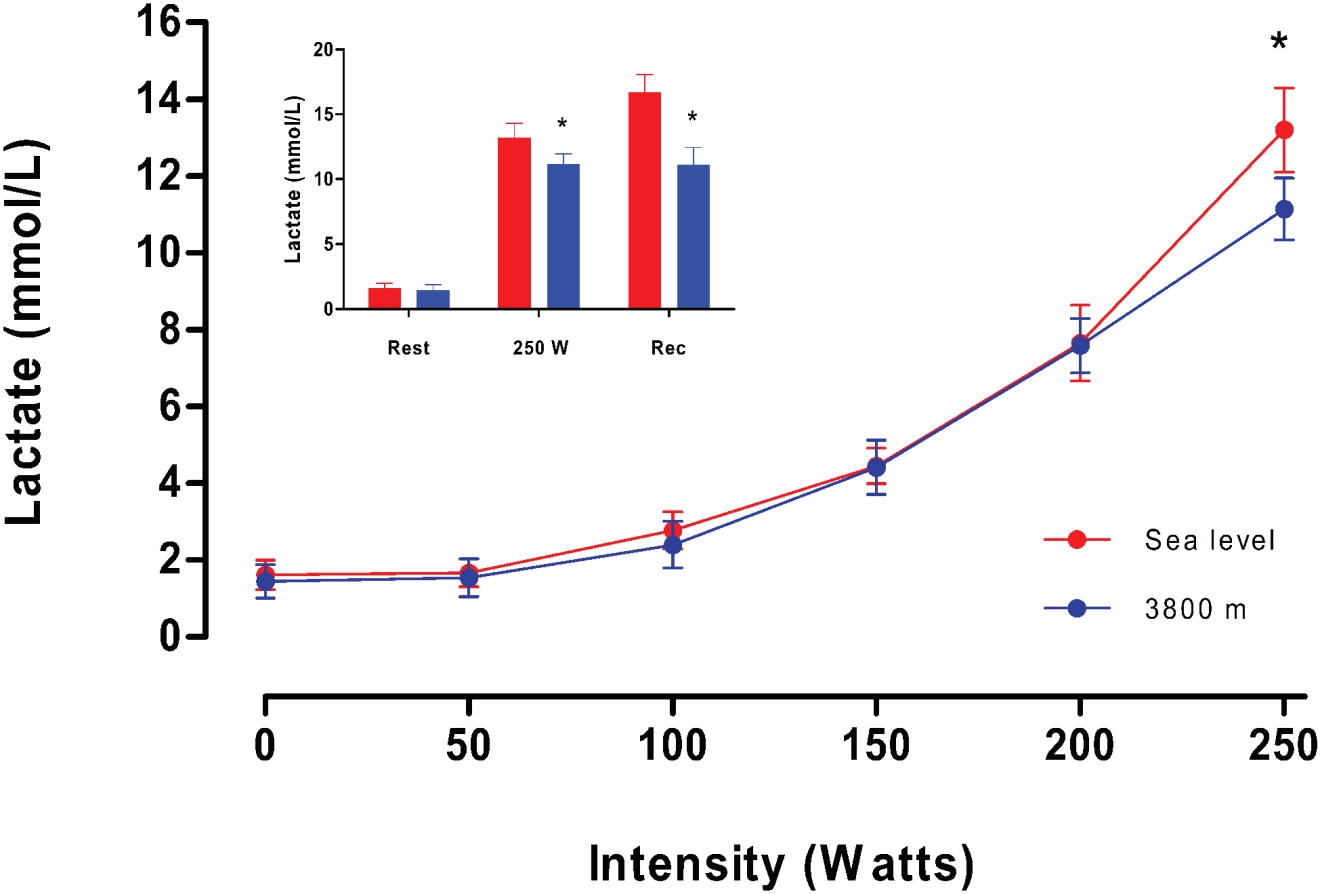
Average values of plasmatic lactate concentration (mmol/L) as a function of intensity (in W) obtained on the fourth day at sea level and high altitude. In the inserted figure, red bars represent the average lactate concentration at Sea level; blue bars are the average lactate concentration (mmol/L) at 3,800 m. * represents a significant difference between sea level vs. high altitude and a cross represents a significant difference compared to rest (*p* < 0.05).

Figure 4 shows plasma [Lac] during maximum exercise at SL or HA. A sustained increase in the mean values of plasma [Lac] is observed with the increase in intensity until 200 W. At maximal effort (250 W), plasma [Lac] in the HA condition was lower than at SL (Table 2; Figure 4). In addition, during the recovery period, we observed that plasma [Lac] in volunteers at SL is higher than that observed at maximal effort (*p* < 0.05, Figure 4). Also, values of plasma [Lac] observed in volunteers at HA are not different to the values described at final maximal effort. Furthermore, a minor [Lac] was observed at HA in comparison with SL (*p* < 0.05, Figure 4).

## Discussion

The present study contributes new data of VO_2_ and plasma [Lac] during maximum exercise at SL and HA conditions in miners acclimatized to CIHH (3,800 m) and for the first time related these variables with work efficiency. Our results show that work capacity (intensity) is maintained despite the fall in oxygen consumption, supporting the idea that there is an increase in work efficiency and a fall in plasma [Lac] during maximal exercise at HA.

A series of experimental protocols were used to investigate the effects of altitude acclimatization. These include the testing of lowlanders (trained or untrained) at SL with simulated altitude or real HA exposure, and testing natives or non-natives after a period of chronic exposure to simulated hypobaric hypoxic conditions or at real altitude conditions, and after deacclimatization (Hochachka et al., 1991; van Hall et al., 2001; Marconi et al., 2006; Calbet and Lundby, 2009). In these studies, both acute and chronic hypoxia had no effect on lowlanders (Bender et al., 1988; Wolfel et al., 1991) nor did they produce an increase (Green et al., 2000) in whole-body steady-state VO_2_ and lactate metabolism during increased exercise intensity (Hochachka et al., 1991; van Hall et al., 2001). However, other studies performed with the same model of exposure to CIHH are scarce.

### Cardiorespiratory and Metabolic Response During Maximum Exercise

In regard to the decreased VO_2_ max (near to 29%) observed in our population at 3,800 m of HA, data observed in the same population at SL are similar to values reported in the literature (Sutton et al., 1988; Calbet et al., 2009). However, this fall in VO_2_ is not related to a fall in intensity (250 W), since in our population intensity was maintained, similar to that described previously by our laboratory at the same altitude at an intensity of 200 W (Moraga et al., 2018). Nowadays, only two studies about aerobic capacity in this model of exposure are available. In the first one, a prospective study was performed in miners with CIHH exposure at 3,800 m, where a decrease in aerobic capacity at an intensity of 175 W was observed, representing a decrease in maximal intensity by approximately 15% after 31 months of CIHH exposure in subjects evaluated at SL (Richalet et al., 2002). However, this study did not report values of maximum VO_2_. In the second study, performed in soldiers exposed to CIHH during 6 months at 3,500 m, included the assessment of VO_2_ max at HA and SL, showing a decreased VO_2_ max (10.6%). However, this article did not report any information about the intensity reached during the test at SL or HA (Prommer et al., 2007).

Previous studies performed at SL on maximum VO_2_ have shown that approximately 10–16% of maximal VO_2_ is used for respiratory muscle work (Aaron et al., 1992; Harms et al., 1998). However, another study where subjects were exposed chronically at 5,260 m supports evidence that 11% of maximal VO_2_ is required for respiratory muscle work during acclimatization (Calbet et al., 2003). In the present study, the assessment of maximal exercise included the measurement of whole body VO_2_ by breath-to-breath methods. In this sense, the lower VO_2_ observed could be due to an increased cost of ventilation at HA. If this response is related to a higher respiratory stimulus at HA, then the lower P_ET_CO_2_ and high values of V_E_/VO_2_ or V_E_/VCO_2_ reported here support this. Similar mechanisms of increased cost of ventilation could explain the response observed in a population of soldiers exposed to CIHH during 6 months (Prommer et al., 2007).

Another possibility that could explain a reduction in VO_2_ max is a reduction in cardiac output, as determined in a series of studies performed in subjects exposed to HA after 9–10 weeks at 5,260 m (Calbet et al., 2003). However, we did not use this work for comparison purposes because of the differences in the experimental condition with our study. Studies performed in populations with this type of exposure showed a decrease in physical performance that was associated with a reduction in the maximal heart rate. This is partially explained by the downregulation of β-adrenergic receptors and upregulation of muscarinic receptors (Richalet et al., 1992), and a detraining effect of exposure to the hypoxia and/or being excessively sedentary (Richalet et al., 2002). Values of maximal heart rate, reported in the study by Richalet et al., are similar to those reported by our group, in subjects with similar exposure times (31 months and over 48 months, respectively). However, values described in the latter study were assessed only at SL, and because we did not observe a depression in heart rate at SL or HA, we can speculate that cardiac output was maintained during our test at SL and at HA.

One remaining explanation for the observed low VO_2_ max could be the reduction in oxygen supply and/or a reduction in oxygen extraction, since it is not determined by a reduction in blood flow to the muscle. Previously, we have demonstrated that in a similar population, with a time exposure of 7–36 months, maximum exercise at 3,800 m and at SL showed a decrease in oxygen saturation, with an increase in hemoglobin concentration and oxygen content calculated unchanged (Moraga et al., 2018). This increase in hemoglobin concentration could be explained by peripheral vasocontraction, which leads to redistribution of blood flow that favors oxygen delivery to the muscle (Lundly et al., 2008). A previous study of chronic exposure during 9 weeks at 5,260 m showed that oxygen extraction is maintained in bike exercise in comparison with acute exposure and normoxia (near 85%), supporting the idea that exercise is not limited by oxygen delivery to the tissue, in this case the muscle (Calbet et al., 2009). Considering that very invasive procedures were not performed in our study, but using the values of venous oxygen saturation described by van Hall et al. (2001), it is possible to estimate the oxygen extraction of our subjects at SL or HA. In this sense, values at rest at SL and HA were similar (44 and 48%, respectively) and at maximal effort no difference were observed (95 and 94%, respectively). If our estimation is adequate, oxygen extraction is higher and maintained at SL or HA (acute, chronic or CIHH), supporting that a possible factor responsible for the reduction in oxygen consumption at HA is given by a redistribution of cardiac output to respiratory muscle during maximal effort.

### Work Efficiency at High Altitude

We previously showed that a minor VO_2_ max was observed at the same intensity at HA compared with SL, and when we calculate the *gross* and *net efficiency* increase with the work intensity at HA, reaching values at a maximal effort of 1.9 and 2.1 times higher compared to SL. In this sense, the present study provides evidence suggesting that workers acclimatized to CIHH have developed acclimation mechanisms that allow them to perform the same work in an environment where oxygen supply is reduced. A similar response was described in an acclimatization study, where an increase in *net efficiency* was reported in a population with prolonged exposure at 6,194 m during 21 days, and suggest that this increase in efficiency at lower VO_2_ max values could be explain by a shift toward a greater glycolytic involvement in ATP regeneration and a preference for carbohydrates over fatty acids during oxidative phosphorylation (Green et al., 2000). Previous studies performed during acclimatization process at 4,300 m support a major dependence of carbohydrate metabolism over fat substrate, supporting that major efficiency is given by a complete oxidation of carbohydrate yields more ATP per more of oxygen than complete oxidation of fats (Brooks et al., 1991; Roberts et al., 1996). Furthermore, another study evaluated the effect of acclimatization on substrate utilization at severe types of exposure to 4,100 m, showing that 70% of substrate used to muscle activity corresponded to carbohydrate oxidation, showing the preference for carbohydrates over fat or protein oxidation at similar exercise intensities (Lundby and Van Hall, 2002). Values of VCO_2_/VO_2_ ratio near of unity are indicative of carbohydrate oxidation (Hochachka et al., 1991), which could imply a reliance on carbohydrates when producing higher ATP yields per mole of O_2_ consumed. Our results complement those previously described with VCO_2_/VO_2_ values of 1.2 at SL and 1.09 at HA, supporting the reliance on carbohydrate metabolism in our subjects at maximal effort (Table 2). Therefore, an enhanced reliance between carbohydrate substrates and ATP production could be explain by the increase in *gross* and *net efficiency* reported in our study, in order to maximize work per mole of ATP consumed. Similar results have shown a higher mechanical efficiency in highland residents, showing that their metabolic and labor efficiency was 1.5 times higher than that of lowlander athletes (Hochachka et al., 1991). Nowadays, all these evidences support that a strong coupling of ATP demand and supply could explain the increased performance of natives with chronic exposure at HAs (Hochachka et al., 1991, 1992; Matheson et al., 1991).

Additionally, we know that repeated resistance training increases aerobic capacity given by an increase in mitochondrial volume density, and in hypoxic conditions, this effect is largely increased (Desplanches et al., 2014). A similar effect was observed in subjects undergoing a training program based on a living high-training low model (Schmutz et al., 2010). In this sense, mitochondrial adjustments to repeated exercise in hypoxia improves the generation of ATP with a reduced oxygen dependency (Green et al., 2009; Brooks, 2018; Chicco et al., 2018). However, subjects that performed a high-intensity intermittent training program in severe hypoxia conditions maintained the same intensity that was observed at SL due to the recruitment of more type II muscle fibers (Amann and Kayser, 2009; Alvarez-Herms et al., 2016). In addition, an anaerobic exercise program showed a negative relationship between oxygen saturation and salivary pH, suggesting an increase in buffer capacity with no increase in plasmatic lactate concentration (Julia-Sanchez et al., 2014), similar to that observed in muscle buffer capacity (Gore et al., 2001). Mizuno et al. (2008) also reported an increase in the muscle buffer capacity in active and less-active subjects that sleep at HA during long exposure periods of 75 days at 5,250 m. In contrast with the previously described, subjects who were repeatedly exposed to 3,550 m during 6 months and performed incremental exercise at SL and at HA, developed acidosis due to an increase in lactate concentration, but showed no modifications in to buffer capacity (Prommer et al., 2007). Considering this, although we did not evaluate pH, buffer capacity, nor ${HCO}_{3}^{-}$ concentration, we estimated the pH values at HA using the Henderson-Hasselbalch equation, P_E_CO_2_ and lactate concentration (Table 2), considering chronic respiratory alkalosis compensation (i.e. reduction of 4 mmol/L in ${HCO}_{3}^{-}$ per each reduction in 10 mmHg in P_E_CO_2_). In this case, we did not observe a difference in pH between SL and HA. If our calculations are correct, we can suggest that the buffer capacity in our population is maintained. However, future studies are necessary to demonstrate this speculative approach.

### Lactate Concentration at High Altitude

Nowadays, studies of the CIHH model that evaluate exercise and measure lactate concentration are scarce. Prior to our study, the only similar article was carried out in a group of soldiers who performed a regular ascent to HA for 11 days, followed by 3 days at SL for 6 months. During the first days of arrival or descent, the maximum oxygen consumption and lactate concentration were evaluated. Under these exposure conditions, maintenance of oxygen consumption and an increase in plasma lactate concentration at HA were reported in subjects exposed to CIHH (Prommer et al., 2007). These results are opposite to those described in the present study, where we demonstrate a decrease in maximum oxygen consumption close to 29% and a decrease in lactate concentration close to 16%, compared to SL. We do not have a complete explanation for these differences between the two studies. However, in a lowland population exposed to an altitude of 5,050 m, a 30% decrease in plasma lactate concentration was observed during maximum effort. Additionally, lactate levels remained low during the first week after descent to SL, about 30%, compared to SL before ascent (Grassi et al., 1996). Furthermore, a lower decrease in plasma lactate concentration (about 21%) was described in subjects exposed to maximum effort at 3,800 m during the first 2 weeks of exposure, reaching a maximum decrease after 8 weeks (close to 31%). Both studies support the presence and permanence of the “lactate paradox” after chronic exposure. As for this study, a decrease in the concentration of plasma lactate was observed in miners of the CIHH model, a response that supports the “lactate paradox”. However, when maximum effort was tested at SL, it was not associated with a decrease in lactate concentration, and the lactate concentration values described in our study are similar to previous works (Grassi et al., 1996; van Hall et al., 2001, 2002, 2009; Pronk et al., 2003; Prommer et al., 2007). This change in plasma lactate concentration values suggests a plastic process that is established after 3 days of resting at SL.

### Limitations

Our study did not perform any invasive evaluation, such as muscle biopsy or lactate metabolism with marked radionuclides, due to pressure from workers’ union and company reasons. Future studies that consider measurements in a larger population of workers and/or the requirements of an animal model are needed to obtain a better understanding of the mechanisms underlying physiological altitude adjustments in the population with exposure to CIHH.

In conclusion, our previous studies showed an almost stable maintenance of oxygen content during a maximal exercise test performed in miners with chronic intermittent exposure to hypoxia at 3,800 m. The present study is the first to show that, in this population, work capacity (intensity) is maintained with a drop in oxygen consumption during a maximum exercise test, which supports that an increase in work efficiency at HA is associated with a lower concentration of lactate.

## Data Availability

The datasets generated for this study are available on request to the corresponding author.

## Ethics Statement

The studies involving human participants were reviewed and approved by Ethics Committee of the Facultad de Medicina of the Universidad Católica del Norte, Chile. The patients/participants provided their written informed consent to participate in this study.

## Author Contributions

FM and JO conceived and designed the study. JO and DJ supervised the overall study. RC-J performed the statistical analysis. DM, RC-J, and JO contributed to sample and data collections. All authors drafted the report and contributed to the interpretation of the results, critical revision of the manuscript, and approval of the final manuscript. FM is the guarantor.

### Conflict of Interest Statement

The authors declare that the research was conducted in the absence of any commercial or financial relationships that could be construed as a potential conflict of interest.
